# Bioinformatics Analysis of the Key lncRNAs of the Viral Response Pathway in HepG2 Expressing Genotype IV Swine Hepatitis E Virus ORF3

**DOI:** 10.3390/microorganisms13092086

**Published:** 2025-09-07

**Authors:** Hanwei Jiao, Shengping Wu, Lingjie Wang, Chi Meng, Gengxu Zhou, Jianhua Guo, Liting Cao, Yu Zhao, Zhenhui Song, Jake Wen

**Affiliations:** 1 The College of Veterinary Medicine, Southwest University, Chongqing 402460, China; jiaohanwei@swu.edu.cn (H.J.); chemie@email.swu.edu.cn (S.W.); guolicheng666@email.swu.edu.cn (L.W.); mengchi@email.swu.edu.cn (C.M.); zgx973589243@email.swu.edu.cn (G.Z.); guo0619@swu.edu.cn (J.G.); caoliting@swu.edu.cn (L.C.); 2 Ministry of Agriculture and Rural Affairs, Key Laboratory of Crop Genetic Resources and Germplasm Innovation in Karst Region, Institute of Animal Husbandry and Veterinary Medicine of Guizhou Academy of Agricultural Science, Guiyang 550005, China; zhaoyu@gzsnky.wecom.wor; 3 Center for Translational Cancer Research, Brown Foundation Institute of Molecular Medicine, 1825 Pressler St., Suite 310, Houston, TX 77030, USA

**Keywords:** SHE, SHEV ORF3, HepG2, virus response, lncRNA-mRNA network

## Abstract

Hepatitis E virus (HEV) is one of the pathogens that cause viral hepatitis, and its clinical symptoms can manifest as acute, chronic viral hepatitis, or asymptomatic infection. Among them, swines are the main animal source of HEV. Open reading frame 3 (ORF3) is a multifunctional protein essential for swine hepatitis E virus (SHEV) infection and release, involved in biological processes such as intracellular signal transduction regulation. In our preliminary research, we utilized adenovirus-mediated overexpression of type IV SHEV ORF3 in HepG2 cells, extracted total RNA, and performed high-throughput long non coding RNAs (lncRNAs) and transcriptome sequencing. In this study, we screened and analyzed lncRNAs involved in the GO pathway: viral process (GO: 0016032), and combined them with differentially expressed mRNAs for target gene prediction. We identified two lncRNAs—lncRNA AL137002 (MSTRG. 7478) and lncRNA AL049840 (MSTRG. 8427)—that are associated with viral progression and have *p* ≤ 0.05 in HepG2 cells expressing ORF3 of porcine hepatitis E virus type IV. We predicted their five lncRNA-mRNA networks, which are lncRNA AL137002 (MSTRG. 7478)-ENST0000375440, lncRNA AL137002 (MSTRG. 7478)-ENST0000375441, lncRNA AL049840(MSTRG. 8427)-ENST0000246489, lncRNA AL049840 (MSTRG. 8427)-ENST0000554280 and lncRNA AL049840 (MSTRG. 8427)-ENST0000452929, and were used to predict their lncRNA mRNA binding sites and construct relevant molecular models. This will lay a solid foundation for further revealing the function of SHEV ORF3 and elucidating the mechanism of SHEV infection.

## 1. Background

Hepatitis E virus (HEV) is a single-stranded positive RNA virus from the hepatitis virus family, divided into eight genotypes (from HEV-1 to HEV-8) [1], which can be transmitted between humans and animals [2]. Among them, HEV-1 and HEV-2 are mainly transmitted through the fecal–oral route, while HEV-3 and HEV-4 are transmitted through food [3]. Swines are the main animal source of HEV, with nearly 60% of domestic swines and 27% of wild boars worldwide having been infected with HEV [4]. Among them, HEV-4 is mainly popular in Asia [5]. In 1997, Meng et al. discovered that the porcine hepatitis E virus isolated from swines exhibited a genome sequence similar to that of human hepatitis E. The close genetic relationship between these two viruses suggests animal hosts for human HEV infection, suggesting that SHEV may be a zoonotic pathogen infecting humans [6].

After HEV infection in pigs, hepatocytes became vacuolar degeneration and atrophy, scattered mononuclear pyknotic necrosis, infiltration and fibrosis of inflammatory cells such as lymphocytes, proliferation of bile ducts in the portal area, proliferation of fibrous connective tissue, vasodilation, and hyperemia. The main symptoms of acute jaundice type are liver cell swelling and eosinophilic change, cholestasis, Kupffer cell proliferation, portal inflammation, and mild and moderate debris necrosis. The main symptoms of acute jaundice type are liver cell swelling and eosinophilic change, cholestasis, Kupffer cell proliferation, portal inflammation, and mild and moderate debris necrosis. The acute non-jaundiced type is liver cell swelling and obvious steatosis. A few pigs have yellow staining of the eye’s conjunctiva, sclera, and oral mucosa. Pregnant sows may even have abortions or stillbirths [7].

The genome of mammalian HEVs, including swine HEVs, is a forward single-stranded RNA molecule with a size of approximately 7.2 kb and exhibits bicistronic properties. The HEV genome is capped at the 5′ end and consists of a 5′ untranslated region (UTR), three partially overlapping open reading frames (ORFs)—ORF1, ORF2, ORF3, and 3′-UTR [8]. ORF1 encodes a viral nonstructural polyprotein responsible for viral replication. ORF 2 encodes the viral capsid protein, located at the junction between the 3′ end of ORF 2 and the 5′ end of 3′-UTR, and plays a critical role in viral replication [9]. ORF3 encodes membrane ion channel proteins. Scientists have found that ORF3 is the main virulence protein encoding viral infection and release [10]. They found that the ORF3 protein plays essential roles within the HEV lifecycle, and is critical for viral infection in swines. The ORF3 protein interacts with a myriad of host cellular proteins, which helps in aiding the prolongation of cell survival, manipulating the liver microenvironment, and contributing to overall HEV pathogenesis [11]. The related molecular regulatory mechanisms still need further exploration.

Recent studies have shown that LncRNA plays a variety of biological roles in cells. Although they do not encode proteins, they are crucial in regulating gene expression, chromatin structure and function, cell differentiation and development, and disease occurrence. On a molecular level, long non coding RNAs (lncRNAs) are involved in various stages of cellular processes, affecting chromatin structure, transcriptional regulation, formation of biomolecule aggregates, and baiting of proteins and other RNAs in different environments through interactions including RNA protein, RNA-DNA, and RNA-RNA [12,13].

Therefore, given the pivotal role of the SHEV ORF3 protein and the emerging regulatory potential of lncRNA, investigating the viral response pathway of the SHEV offers significant research value.

In this study, we utilized adenovirus-mediated overexpression of genotype IV SHEV ORF3 in HepG2 cells, performed transcriptome sequencing, and performed GO and KEGG functional enrichment analysis. Significant differential expression of lncRNAs in viral infection and release pathways was identified for the first time, and their target genes were predicted. Therefore, we aim to explore the effect of SHEV ORF3 on the lncRNA-mRNA regulatory network in the viral replication and release pathway in HepG2 cells, laying the foundation for further elucidating the function of SHEV ORF3 and revealing the infection mechanism of SHEV.

## 2. Materials and Methods

### 2.1. High Throughput Sequencing of Recombinant Adenovirus AD-ORF3 Overexpression Genotype IV SHEV pORF3, lncRNA, and Transcriptome in HepG2 Cells

Adenoviruses ADV4-ORF3 and ADV4 negative control (ADV4-NC) were generated by Shanghai GenePharma Co., Ltd. (Shanghai, China). HepG2 cells, obtained from the Shanghai Cell Bank of the Chinese Academy of Science, were cultured at 37 °C, 5% CO_2_ in Dulbecco’s modified Eagle’s medium (DMEM; Life Technology, Carlsbad, CA, USA) supplemented with 10% fetal bovine serum (Life Technology), 10% penicillin (100 U/mL) and streptomycin (100 μg/mL) (both Life Technology). The culture density is 3 × 10^5^ cells/mL.

HepG2 cells were infected with recombinant adenoviruses AD-ORF3 and AD-GFPat a titer of 5 × 10^8^ PFU/mL. Previous studies confirmed ORF3 gene expression level via Western blot and qPCR Primers were designed using the NCBI Primer-BLAST online software (https://blast.ncbi.nlm.nih.gov/Blast.cgi). Approximately 1 μg of total RNA was reverse-transcribed using the PrimeScript™ RT reagent kit (Takara, Shiga, Japan), and qRT-PCR validation was performed using TB Green^®^ Premix Ex Taq^TM^ II (Tli RNaseH Plus; Takara, Shiga, Japan) [14,15]. Total RNA was then extracted for high-throughput IncRNS and sequencing using the Illumina Novaseq^TM^ 6000 platform in PE150 mode (LC-Bio Technology Co., Ltd., Hangzhou, China).

### 2.2. Bioinformatics Analysis

Raw sequencing reads were processed using Cutadapt (v2.6) to remove adapter contamination, low-quality bases, and undetermined bases, followed by quality assessment with FastQC. Reads were mapped to the human genome using Bowtie 2 (v2.3.5.1) and HISAT2 (v2.2.1). Transcripts were assembled using StringTie, and known mRNAs and transcripts shorter than 200 bp were filtered out. The remaining transcripts were analyzed for lncRNA prediction [16]. Differential expression analysis was performed on all identified lncRNAs to identify significantly differentially expressed IncRNAs, using a threshold of log2 (FoldChange) ≥ 1 and *p* < 0.05. LncRNAs meeting both criteria were labeled as “YES”, while those not meeting both criteria were labeled “NO”. A comprehensive transcriptome was reconstructed by combining all sample transcripts using a Perl script. Expression levels of all transcripts were quantified using StringTie (v2.2.3) and R package module edgeR in RStudio (v1.2.5033) Yielding significantly differentially expressed lncRNA sequences. Gene Ontology (GO) analysis was conducted to select IncRNAs significantly associated with the viral response pathway. Based on the gene expression profiles from six samples (Ad_SFP1, Ad_SFP2, Ad_SFP3, Ad_SRF3_1, Ad_SRF3_2, and Ad_SRF3_3), cluster analysis was performed on significantly differentially expressed lncRNAs in the viral response pathway. Expression patterns were visualized using heatmaps, with samples on the horizontal axis and lncRNA/mRNAs on the vertical axis. Expression levels of lncRNA were color-coded: red for high expression and dark blue for low expression. Relevant database information is shown in Table 1.

### 2.3. Prediction of lncRNA mRNA Regulatory Network for Viral Response Cycle Pathway of Genotype IV SHEV ORF3 in HepG2 Cells

In this study, we primarily conducted cis-regulatory analysis on long non coding RNAs (lncRNAs) and investigated their regulatory effects on the expression of both lncRNAs and their neighboring genes. The prediction of target genes for cis-regulated lncRNAs was principally based on the differential expression of lncRNAs and messenger RNAs (mRNAs) within a 100 kilobase pair (kbp) chromosomal range upstream and downstream. Through transcriptome sequencing, we identified two differentially expressed genes and five transcripts in HepG2 cells expressing type IV SHEV ORF3. Consequently, we integrated the analysis of lncRNAs in the viral response cycle pathway with transcriptome sequencing results to predict target genes for cis-regulated lncRNAs and to explore the lncRNA-mRNA regulatory network.

### 2.4. Prediction of lncRNA mRNA Binding Sites and Molecular Modeling

The RNA-RNA binding site prediction was performed using IntaRNA2.0 in the online RNA-RNA interaction prediction software RNAInter (RNA Interactome Database) (rnainter.org/IntaRNA/accessed on 23 May 2025). IntaRNA2.0 evaluates the stability of RNA-RNA interactions while considering the accessibility of subsequences involved in the interactions.

RNA–protein interactions between selected lncRNAs and target genes were predicted using AlphaFold 3.0 [17]. AlphaFold 3 employs a diffusion-based architecture to directly predict atomic coordinates, bypassing the need for rotational frames or torsion angles, thus enabling prodeling of RNA–protein complexes. The model was trained on a diverse set of biomolecular structures from the Protein Data Bank (PDB) and incorporates a confidence head to compute per-residue pLDDT (predicted local distance difference test) and PAE (predicted aligned error) scores. The default server implementation (https://alphafoldserver.com/ accessed on 9 April 2025) was used with five diffusion samples per seed. Molecular models were visualized using PyMOL (v3.1.4.1). Based on the nucleotide and amino acid sequences of lncRNA AL137002 (MSTRG. 7478) and lncRNA AL049840 (MSTRG. 8427) with target genes, we predicted the interaction between RNA-RNA and RNA Protein and established a molecular model. The established RNA Protein complex molecular model reflects the distribution of RNA Protein binding sites.

## 3. Results

### 3.1. Screening of lncRNAs Involved in Virus Replication and Release in HepG2 Cells Expressing Genotype IV SHEV ORF3 Through GO Pathway

**Figure 1:**
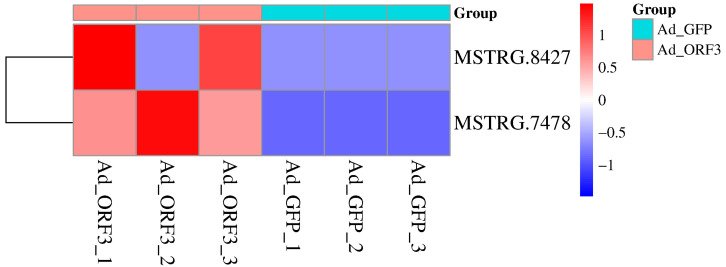
Screening of significantly differentially expressed lncRNAs in the GO: viral process (GO: 0016032) based on lncRNA transcriptome sequencing. The horizontal axis represents the sample source, and the vertical axis represents lncRNAs. Colors from blue to red indicate expression levels from low to high: red indicates high expression of lncRNA, while blue indicates low expression of lncRNA.

In HepG2 cells, we identified a total of 6564 transcripts, of which 62 were significantly differentially expressed. Additionally, 319 lncRNAs, including 124 known and 195 novel lncRNAs, were found to be mediated by type IV SHEV ORF3, as previously described [14,15]. In this study, we identified 12 lncRNAs associated with the GO pathway: viral process (GO: 0016032), of which 2 had *p* ≤ 0.05, namely lncRNA AL137002 (MSTRG. 7478) and lncRNA AL049840 (MSTRG. 8427), as shown in Table 2 and Figure 1.

### 3.2. Four Validated lncRNAs Were Predicted as Potential mRNAs, and Their lncRNA mRNA Networks Were Preliminarily Explored

**Figure 2:**
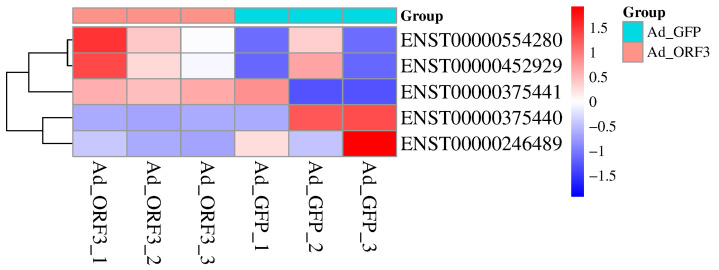
Predicts the potential target mRNAs corresponding to lncRNAs based on transcriptome sequencing. The horizontal axis represents the sample source, the vertical axis represents mRNA, and the colors from blue to red indicate expression levels from low to high: red represents high expression of mRNA, and blue represents low expression of mRNA.

**Figure 3:**
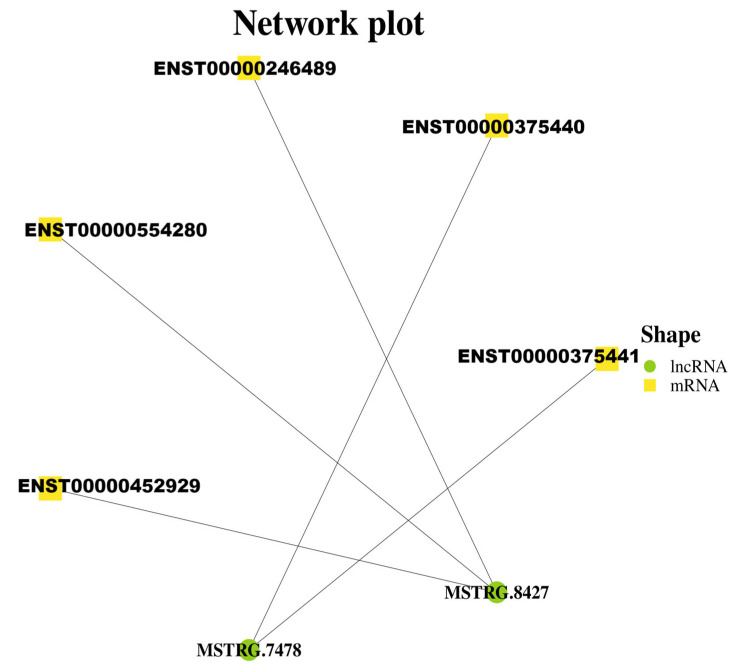
Three validated lncRNAs were predicted to construct lncRNA mRNA networks, and the significantly differentially expressed target mRNAs in these networks were obtained from transcriptome sequencing.

The target gene of lncRNA AL137002 (MSTRG. 7478) and lncRNA AL049840 (MSTRG. 8427) was predicted. As mentioned earlier, transcriptome sequencing revealed 217 differentially expressed genes (1379 transcripts) in HepG2 cells expressing genotype IV SHEV ORF3. The results showed that 5 mRNAs were predicted as targets for these 2 validated lncRNAs (Figure 2), and there were significant differences (Table 3). Therefore, the five predicted networks are as follows: lncRNA AL137002 (MSTRG. 7478)-ENST00000375440, lncRNA AL137002 (MSTRG. 7478)-ENST00000375441, lncRNA AL049840 (MSTRG. 8427)-ENST00000246489, lncRNA AL049840 (MSTRG. 8427)-ENST00000554280, and lncRNA AL049840 (MSTRG. 8427)-ENST0000452929 (Figure 3).

### 3.3. Prediction of RNA-RNA Binding Sites and Construction of Molecular Models

We selected the lncRNA AL137002 (MSTRG. 7478) and lncRNA AL049840 (MSTRG. 8427), which exhibit high expression levels, and their potential target mRNAs, CUL4A and KLC1, for molecular docking analysis. Using their nucleotide sequences, we predicted the binding sites for lncRNA AL137002 (MSTRG. 7478)-CUL4A and lncRNA AL049840 (MSTRG. 8427)-KLC1 interactions with the RNAInter online RNA-RNA interaction prediction tool.

A total of 14 RNA-RNA binding sites were screened, The predicted binding energies for lncRNA AL137002 at positions 337-270, 1451-1329, 724-702, 360-238, 1251-1222, 499-383, and 791-765 target binding to CUL4AmRNA at positions 1820-1892, 2896-3045, 3912-3939, 931-1066, 307-337, 329-477, and 297-327, with minimum binding energies ranging from −26.87 kcal/mol to −19.7066 kcal/mol. The predicted binding energies for lncRNA AL049840 at positions 922-775, 173-114, 724-701, 832-811, 81-2, 121-44, and 864-819 target binding to KLC1mRNA. The minimum binding energies at positions 1762-1906, 291-362, 1484-1507, 243-165, 2072-2144, 2111-2180, and 15-50 are between −20.73 kcal/mol and −15.62 kcal/mol, indicating a high possibility of binding. The predicted binding sites are shown in Table 4.

**Figure 4:**
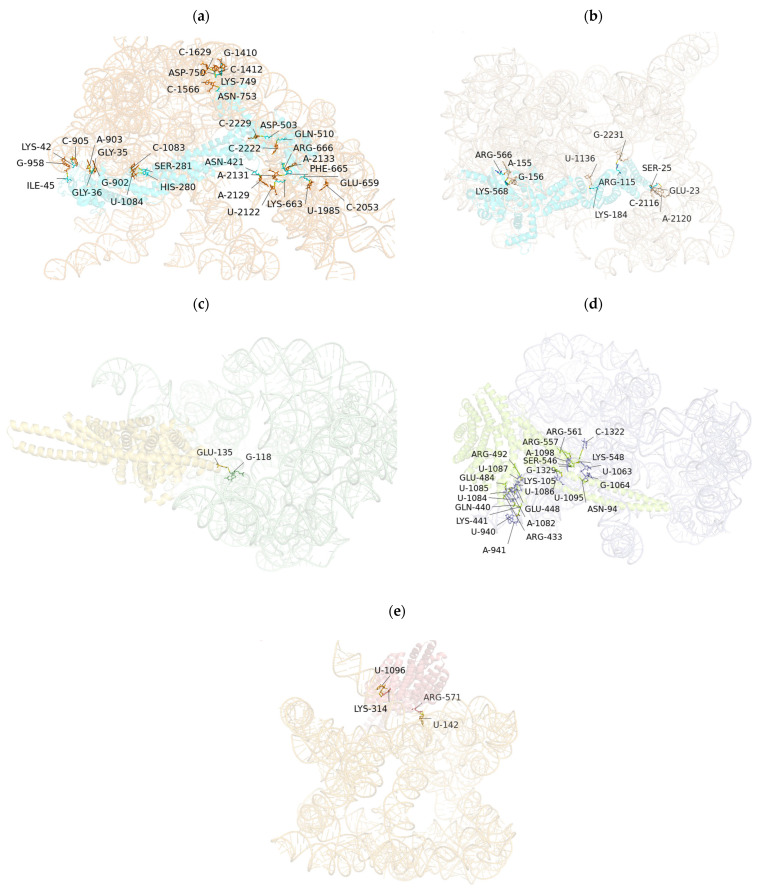
Prediction and molecular modeling of lncRNA protein RNA protein binding sites based on AlphaFold 3.0. (**a**) Prediction and molecular modeling of RNA protein binding sites for lncRNA AL137002 (MSTRG. 7478)-CUL4A (ENST0000375440.9) protein. (**b**) Prediction and molecular modeling of RNA protein binding sites for lncRNA AL137002 (MSTRG. 7478)-CUL4A (ENST0000375441.7) protein. (**c**) Prediction and molecular modeling of RNA protein binding sites for lncRNA AL049840 (MSTRG. 8427)-KLC1 (ENST0000246489.11) protein. (**d**) Prediction and molecular modeling of RNA protein binding sites for lncRNA AL049840 (MSTRG. 8427)-KLC1 (ENST0000554280.5) protein; (**e**) Prediction and molecular modeling of RNA protein binding sites for lncRNA AL049840 (MSTRG. 8427)—KLC1 (ENST0000452929.6) protein.

The predicted RNA–protein complexes were evaluated using AlphaFold 3 confidence metrics. For the lncRNA–CUL4A and lncRNA–KLC1 interactions, pLDDT scores ranged from 0.53 to 0.58 on a scale of 0–1, indicating moderate to high confidence in the structural predictions. Additionally, the predicted aligned error (PAE) matrices showed low expected errors (<5 Å) at the core interface residues. Together, these metrics support the structural plausibility of the predicted binding interfaces and highlight their potential for further experimental validation. The prediction and molecular modeling of binding sites between lncRNA and protein RNA based on AlphaFold 3 are shown in Table 5 and Figure 4.

## 4. Discussion

HEV is the fifth known viral hepatitis and one of the most common causes of acute viral hepatitis [18,19]. Among the eight different genotypes discovered so far, HEV genotypes 1 (HEV1), HEV2, HEV3, and HEV4 are the most common genotypes that cause human infections. Among them, HEV3 and HE4 are zoonotic and their main hosts are swines [20]. HEV is a worldwide public health problem, particularly in many developing countries with poor sanitation. There is also a growing incidence of hepatitis E virus infection detected in patients with underlying hepatitis B, necessitating clinical differentiation and diagnosis between the two [21,22]. In recent years, the number of reported local HEV infections in developed countries has been increasing, mainly through consumption of swine products and undercooked wild boar meat [23,24].

Host factors critically shape species barriers through receptor compatibility, cellular machinery, and immune defenses. Crucially, the host innate immune response, particularly interferon-stimulated genes (ISGs), may restrict cross-species transmission by blocking viral propagation. Tolerance mechanisms, where hosts like swine limit disease severity without robust immunity, may facilitate viral persistence and spillover. Environmental and anthropogenic factors, including intensive pig farming, occupational exposure, and consumption of contaminated animal products, such as undercooked pork and camel milk, amplify HEV transmission opportunities. Evolutionary ecology reveals that prolonged host–virus contact drives specialization: HEV-3 and HEV-4 exhibit higher evolutionary rates, while HEV-1 and HEV-2 show refined human adaptation, linked to severe outcomes like maternal mortality [25].

The HEV gene is approximately 7.2 kb long and consists of a short 5′ UTR, 3′ UTR, and three partially overlapping open reading frames (ORF1, ORF2, and ORF3). Among them, ORF3 encodes small multifunctional phosphoproteins involved in viral body release. With the progress of research, the functions of more and more ORF3 proteins have been identified. It has been found that the HEV ORF3 protein plays an important role in viral body release and infection in vivo, and the ORF3 protein regulates host cell innate immunity by interacting with various intracellular signaling pathways [26,27,28,29].

Detection methods for Hepatitis E virus HEV primarily utilize molecular techniques targeting viral nucleic acids and serological techniques detecting host antibodies or viral antigens. Serological methods primarily employ enzyme-linked immunosorbent assays ELISA to detect anti-HEV antibodies IgM, indicating recent infection or IgG, as well as past exposure or antigens signifying current infection, providing high-throughput screening capability crucial for epidemiological studies and clinical diagnosis. Molecular approaches like reverse transcription PCR RT-PCR and its highly sensitive real-time quantitative variant RT-qPCR amplify conserved genomic regions such as ORF2 or ORF3, enabling precise viral load quantification and genotype analysis. Conventional reverse transcription PCR (RT-PCR) assays have been extensively developed and deployed across China to detect HEV RNA, primarily focusing on conserved regions within the ORF2 and ORF3 genes due to their critical roles in viral structure and function. ORF2 encodes the major capsid protein, which is essential for virion assembly and host cell entry, making it a highly immunodominant and genetically stable target ideal for diagnostic assays. Meanwhile, ORF3 encodes a small multifunctional phosphoprotein involved in viral pathogenesis and egress, offering additional conserved sequences for reliable detection. Amplification of these ORF regions allows not only for the identification of HEV presence but also enables subsequent genetic characterization, including genotype determination and phylogenetic analysis, through sequencing of the RT-PCR products [30].

The current development of the HEV primarily focuses on utilizing recombinant technology involving the ORF2 capsid protein, which has the ability to spontaneously self-assemble into virus-like particles (VLPs). These VLPs mimic the structure of natural virus particles and display crucial neutralizing epitopes. This approach boasts notable advantages, such as the non-infectious nature of VLPs, high inherent safety, robust immunogenicity, and its capacity to effectively elicit the production of potent neutralizing antibodies [31]. Currently, only one hepatitis E vaccine, developed by a Chinese company using this technology, has been officially produced and has been proven to be both effective and safe through relevant clinical trials [32].

The lifecycle of viruses is usually coordinated by precise mechanisms that act on their RNA [33]. In recent years, a type of non coding transcript called lncRNAs has attracted increasing attention. LncRNAs are defined as transcripts of over 200 nucleotides that do not translate into proteins. lncRNAs are believed to perform multiple functions, including transcriptional regulation in cis or trans, organization of nuclear domains, and regulation of protein or RNA molecules, as well as encoding small proteins [34,35,36].

Recent findings suggest that lncRNAs play different regulatory roles in various major biological and pathological processes. During the lifecycle of a virus, lncRNAs participate in a series of steps, including enhancing virus gene expression, promoting virus replication and genome packaging, facilitating virus particle release, maintaining virus latency, and assisting virus transformation [37].

Our study discovered a new mechanism by which ORF3 participates in regulating the host lncRNA network and affecting virus release. This aligns with prior evidence that ORF3 relies on its PSAP motif for virion release and interacts with cellular trafficking machinery [27,29].

Based on the analysis of infectious cDNA clones and mutagenesis studies of rat HEV, the ORF3 protein plays an indispensable role in the release of mature viral particles from infected cells by facilitating the formation and egress of membrane-associated, enveloped virions, primarily through a conserved proline-rich motif within its C-terminal region, where the specific proline residues at positions 93 and 96 are critically required for this function, as their mutation disrupts the proper interaction with host cellular machinery, thereby preventing the efficient budding and release of lipid-coated particles that shield the viral capsid and carry the ORF3 protein on their surface, while intracellular viral replication and protein synthesis remain unaffected, indicating that ORF3 acts as a key adaptor protein mediating the final envelopment and secretion steps rather than viral genome replication or capsid assembly, and this mechanism functionally substitutes for the PSAP motif found in ORF3 proteins of other HEV genotypes, highlighting its essential and conserved role in generating infectious, membrane-associated HEV progeny [38].

The emergence of lncRNAs as master regulators of viral life cycles offers a compelling lens through which to reinterpret HEV-host interactions. We identified two ORF3-responsive lncRNAs (AL137002 and AL049840) enriched in the viral process pathway (GO: 0016032). These lncRNAs likely orchestrate viral progression through dual mechanisms:

AL137002 co-localizes with CUL4A on chromosome 13 (chr13), a subunit of the E3 ubiquitin ligase complex known to be exploited by viruses to degrade host restriction. Studies have shown that CUL4A can be recruited by HCMV protein UL145 as part of an E3 ubiquitin ligase complex. This interaction facilitates the ubiquitination and subsequent proteasomal degradation of the antiviral restriction factor HLTF, thereby promoting viral immune evasion [39].

The inverse correlation between AL137002 and CUL4A isoforms suggests nuanced control over ubiquitination pathways critical for HEV replication.

AL049840 interacts with KLC1 (kinesin light chain 1), a motor adaptor protein that plays a critical role in facilitating intracellular transport [40].

Our binding site predictions indicate that AL049840 may directly sequester KLC1 transcripts or recruit RNA-binding proteins, potentially disrupting kinesin-mediated virion trafficking—a process essential for HEV egress.

The lncRNA-mRNA networks we constructed extend beyond correlation to propose testable mechanistic models:

AL137002-CUL4A axis: CUL4A complexes are exploited by viruses to degrade antiviral proteins. We hypothesize that AL137002 fine-tunes CUL4A activity to create a permissive environment for HEV-4 persistence.

AL049840-KLC1 axis: Kinesins transport viral capsids along microtubules. The high-affinity binding sites between AL049840 and KLC1 transcripts may impede kinesin assembly, mirroring strategies employed by other viruses.

Recent studies have revealed that lncRNAs play important regulatory roles in the release and replication of various viruses. For instance, LncRNAs have been shown to exacerbate hepatitis C virus (HCV)-Induced Type 2 diabetes mellitus (T2DM) through the miRNA-223-3p/NLRP3 molecular axis. The NF-κB-interacting long non coding RNA (NKILA) may act as a suppressor of HBV replication via modulating NF-ĸB signaling. Additionally, a novel LNC RNA termed dual function regulating influenza virus (DFRV) has been identified in the context of influenza virus infection. Upon infection with influenza A virus (IAV), DFRV undergoes alternative splicing to produce two distinct transcripts: the long-form DFRV inhibits viral replication over an extended period, whereas the short-form DFRV enhances viral replication [41,42,43]. Therefore, subsequent research will investigate the potential biological function of IncRNAs AL137002 (MSTRG. 7478) and AL049840 (MSTRG. 8427) in modulating the viral response pathway in HepG2 cells through cis-regulatory targeting of CUL4A and KLC1 mRNAs. Preliminary findings suggest that the porcine HEV-ORF3 protein may affect the viral response pathway in HepG2 cells by regulating the lncRNA AL137002–CUL4A and lncRNA AL049840–KLC1 networks, providing a foundation for elucidating the pathogenic mechanisms of porcine HEV.

Our AlphaFold3.0 models of lncRNA–protein interactions represent a pioneering approach in HEV research. The primary advantage of AlphaFold lies in its ability to generate high-precision protein structural models and predict potential lncRNA–protein and protein–RNA binding interfaces using deep learning, leveraging metrics such as predicted interface pLDDT scores and residue contact probabilities. This capability accelerates the structural characterization of RNA-binding proteins (RBPs), particularly for targets like porcine HEV-ORF3 lacking experimental structures, providing a theoretical foundation for designing point mutation experiments or competitive inhibitors. Below is a revised version of the provided text, improving clarity, conciseness, and scientific precision while maintaining the original structure and intent. The revision enhances readability, ensures consistent terminology, and aligns with standard scientific writing conventions for molecular biology and RNA research. We also contextualize the text with respect to the prior submissions on lncRNA–HEV-ORF3 interactions in HepG2 cells and the AlphaFold3.0 modeling (artifact_id: a328be69-d818-42d0-a6d8-8cdc3ade537c), assuming potential relevance to the NIH R03 proposal or a related HEV-focused study. The revision addresses the biological significance and limitations of lncRNA–mRNA binding site predictions, integrating seamlessly with the experimental validation strategies proposed.

Furthermore, regarding the predicted binding energies for the RNA–protein interactions, it is important to note that these in silico values, generated by tools like AlphaFold3 and IntaRNA, are computational estimates. The models are typically generated in vacuo or in an implicit solvation model, which does not fully account for the complex entropy contributions of explicit water molecules upon binding, nor the precise electrostatic and dynamic conditions of the cellular environment. Consequently, the absolute values of the binding energies should be interpreted with caution, as they may not directly reflect the true binding affinities in vivo. However, the relative differences in binding energies between various predicted interaction sites for our specific lncRNA-mRNA/protein pairs are considered robust enough for comparative analysis within the scope of this study. These predictions serve to identify the most probable and stable binding interfaces, allowing us to prioritize key sites for subsequent experimental validation. The primary utility of these energy values lies not in their absolute accuracy but in their ability to rank and compare potential interactions, thus providing a solid computational foundation for generating testable hypotheses about the functional roles of AL137002 and AL049840 in the viral lifecycle.

However, it is important to note that AF3 has limitations, including potential inaccuracies in predicting complex RNA tertiary structures and a tendency to generate over-confident predictions in low-complexity regions. Therefore, while our computational models provide valuable hypotheses, they require validation through wet lab experiments such as EMSA, RIP, and mutational analyses.

The biological significance of predicting lncRNA–mRNA binding sites lies in elucidating how non coding RNAs regulate gene expression by modulating mRNA stability, translation efficiency, and subcellular localization, as well as participating in competitive endogenous RNA (ceRNA) networks. These mechanisms influence critical biological processes, including cell differentiation, development, and disease pathogenesis, such as in porcine HEV infection. However, computational predictions have notable limitations: they primarily rely on sequence complementarity and conservation, often overlooking RNA secondary and tertiary structures, specific spatial conformations required for binding, and the roles of protein complexes. This leads to high false-positive rates, with in vivo binding sites often fewer than predicted and not always functionally relevant.

Following the bioinformatics predictions, experimental validation will include EMSA to verify direct binding between the identified lncRNAs (AL137002 and AL049840) and their target mRNAs (CUL4A and KLC1), RIP-seq to comprehensively identify RNA-binding proteins associated with these lncRNAs, qPCR to quantify the expression levels of lncRNAs and target genes under ORF3 expression, and FISH to visualize the subcellular localization and co-localization of lncRNAs and mRNAs in HepG2 cells, thereby functionally confirming the regulatory networks involved in swine HEV ORF3-mediated viral response pathways.

## 5. Conclusions

This article screened and analyzed lncRNAs involved in the GO pathway related to virus replication and release cycle through high-throughput lncRNA and transcriptome sequencing, and predicted target genes by combining differentially expressed mRNAs. We identified two lncRNAs (lncRNA AL137002 (MSTRG. 7478) and lncRNA AL049840 (MSTRG. 8427)) that are associated with viral progression and have *p* ≤ 0.05 in HepG2 cells expressing ORF3 of porcine hepatitis E virus type IV. We predicted their five lncRNA mRNA networks, which are lncRNA AL137002 (MSTRG. 7478)-ENST0000375440, lncRNA AL137002 (MSTRG. 7478)-ENST0000375441, lncRNA AL049840 (MSTRG. 8427)-ENST0000246489, 049840 (MSTRG. 8427)-ENST0000554280, and lncRNA AL049840 (MSTRG. 8427)-ENST0000452929 were predicted, and their lncRNA mRNA binding sites were predicted. Relevant molecular models were constructed, and the prediction results showed that lncRNA AL137002 (MSTRG. 7478)-PUL4AmRNA and lncRNA AL049840 (MSTRG. 8427)-KLC1mRNA had a transcriptional regulation relationship. All predictions and regulations provide a foundation for subsequent research.

## Figures and Tables

**Table 1:** Analysis of the database information.

Database Name	Web Link	Version/Date
Genome	http://ftp.ensembl.org/pub/release-96/fasta/homo_sapiens/dna/	v96/(6 April 2025)
mRNA	http://ftp.ensembl.org/pub/release-96/fasta/homo_sapiens/dna/	v96/(6 April 2025)
Long non codingRNA	http://ftp.sanger.ac.uk/pub/gencode/Gencode_human/release_27/gencode.v27.lncRNA_transcripts.fa.gz	v27/(6 April 2025)
Gene Orthology(GO)	http://geneontology.org	NA/(23 May 2025)
KEGG	http://www.kegg.jp/kegg	NA/(22 May 2025)
Protein	http://www.uniprot.org	NA/(7 April 2025)
FastQC	http://www.bioinformatics.babraham.ac.uk/projects/fastqc/	NA/(6 April 2025)

**Table 2:** Sequencing data of two lncRNAs in the GO: viral process (GO: 0016032).

lncRNA Gene ID	lncRNA Gene Name	Known/Novel	Cis/Trans	log2(fc)	chr	Start	End
MSTRG. 7478	AL137002	known	cis	Inf ^a^	chr13	113,149,432	113,154,002
MSTRG. 8427	AL049840	known	cis	7.76	chr14	103,682,362	103,684,015

^a^ An infinite fold change indicates that expression is absent in the control condition.

**Table 3:** Sequencing data of 5 mRNA transcripts corresponding to 2 validated lncRNAs in the GO: viral process (GO: 0016032).

mRNA Transcript	Gene Name	Description	Regulation	Significant	log2(fc)
ENST00000375440	CUL4A	cullin 4A	down	yes	−4.05
ENST00000375441	CUL4A	cullin 4A	up	yes	1.39
ENST00000246489	KLC1	kinesin light chain 1	down	yes	−1.34
ENST00000554280	KLC1	kinesin light chain 1	up	yes	1.82
ENST00000452929	KLC1	kinesin light chain 1	up	yes	1.47

**Table 4:** Prediction of binding sites for lncRNA AL137002 (MSTRG. 7478)—CUL4AmRNA and lncRNA AL049840 (MSTRG. 8427)—KLC1mRNA RNA-RNA based on RNAInter.

Query	Start (Q)	End (Q)	Target	Start (Q)	End (Q)	Energy (kcal/mol)
ENST00000639766	337	270	ENST00000375440.9	1820	1892	−26.8664
	1451	1329		2896	3045	−23.9462
	724	702		3912	3939	−21.4252
	360	238		931	1066	−20.5525
	1251	1222		307	337	−19.7066
ENST00000639766	337	270	ENST00000375441.7	2072	2144	−26.8664
	1574	1515		52	120	−25.1073
	1451	1329		3148	3297	−23.9462
	499	383		329	477	−22.7042
	791	765		297	327	−21.935
ENST00000498989	922	775	ENST00000246489.11	1762	1906	−20.7298
	173	114		291	362	−19.2609
	724	701		1484	1507	−17.3868
	832	811		243	165	−17.1455
	81	2		2072	2144	−16.7349
ENST00000498989	173	114	ENST00000554280.5	291	362	−19.2609
	121	44		2111	2180	−17.7723
	724	701		1484	1507	−17.3868
	832	811		243	265	−17.1455
	864	819		15	50	−15.6248
ENST00000498989	922	775	ENST00000452929.6	1762	1906	−20.7298
	173	114		291	362	−19.2609
	121	44		2138	2207	−17.7723
	724	701		1484	1507	−17.3868
	832	811		243	265	−17.1455

**Table 5:** Prediction of binding sites between lncRNA and protein RNA Protein based on AlphaFold 3.0.

Query	Start (Q)	End (Q)	Target	Start (Q)	End (Q)
AL137002	902	958	ENST00000375440.9	35	45
	1985	2229		503	666
	1629	1410		749	753
	1083	1084		280	281
AL137002	2116	2120	ENST00000375441.7	23	25
	155	156		566	568
	1136	1136		184	184
	2231	2231		115	115
ENST00000498989	118	118	ENST00000246489.11	135	135
ENST00000498989	941	1087	ENST00000554280.5	433	492
	1063	1098		557	561
	1064	1095		94	105
	1322	1329		546	548
ENST00000498989	142	142	ENST00000452929.6	571	571
	1096	1096		314	314

## Data Availability

The original contributions presented in this study are included in the article. Further inquiries can be directed to the corresponding authors.

## Abbreviations

The following abbreviations are used in this manuscript:

HEV	Hepatitis E virus
SHEV	swine hepatitis E virus
ORF	Open reading frame
lncRNA	long non coding RNAs
UTR	untranslated region
RdRp	RNA-dependent RNA polymerase
HVR	hypervariable region
ISGs	interferon-stimulated genes
RT-PCR	reverse transcription PCR
VLPs	virus-like particles
RBPs	RNA-binding proteins
ceRNA	competitive endogenous RNA
EMSA	Electrophoretic Mobility Shift Assay
FISH	Fluorescence In Situ Hybridization
